# Human Norovirus Cultivation in Nontransformed Stem Cell-Derived Human Intestinal Enteroid Cultures: Success and Challenges

**DOI:** 10.3390/v11070638

**Published:** 2019-07-11

**Authors:** Mary K. Estes, Khalil Ettayebi, Victoria R. Tenge, Kosuke Murakami, Umesh Karandikar, Shih-Ching Lin, B. Vijayalakshmi Ayyar, Nicolas W. Cortes-Penfield, Kei Haga, Frederick H. Neill, Antone R. Opekun, James R. Broughman, Xi-Lei Zeng, Sarah E. Blutt, Sue E. Crawford, Sasirekha Ramani, David Y. Graham, Robert L. Atmar

**Affiliations:** 1 Department of Molecular Virology and Microbiology, Baylor College of Medicine, Houston, TX 77030, USA; 2 Department of Medicine, Gastroenterology and Hepatology, Baylor College of Medicine, Houston, TX 77030, USA; 3 Department of Medicine, Infectious Diseases, Baylor College of Medicine, Houston, TX 77030, USA; 4 Department of Virology II, National Institute of Infectious Diseases, Musashi-murayama, Tokyo 208-0011, Japan

**Keywords:** human norovirus cultivation, intestinal enteroids/organoids, virus neutralization and inactivation

## Abstract

Noroviruses, in the genus *Norovirus*, are a significant cause of viral gastroenteritis in humans and animals. For almost 50 years, the lack of a cultivation system for human noroviruses (HuNoVs) was a major barrier to understanding virus biology and the development of effective antiviral strategies. This review presents a historical perspective of the development of a cultivation system for HuNoVs in human intestinal epithelial cell cultures. Successful cultivation was based on the discovery of genetically-encoded host factors required for infection, knowledge of the site of infection in humans, and advances in the cultivation of human intestinal epithelial cells achieved by developmental and stem cell biologists. The human stem cell-derived enteroid cultivation system recapitulates the multicellular, physiologically active human intestinal epithelium, and allows studies of virus-specific replication requirements, evaluation of human host-pathogen interactions, and supports the pre-clinical assessment of methods to prevent and treat HuNoV infections.

## 1. Introduction

Human noroviruses (HuNoVs) are the leading cause of epidemic and sporadic acute gastroenteritis worldwide and are the primary cause of food-borne gastroenteritis [1,2,3,4,5,6]. Since the introduction of rotavirus vaccines for children and the overall decrease in rotavirus illness, HuNoVs have become the predominant gastrointestinal pathogen within pediatric populations in developed countries [7,8]. HuNoVs are highly contagious, with rapid person-to-person transmission through the fecal-oral route and indirectly from contact with contaminated fomites or consumption of contaminated food or water. In addition to causing morbidity and mortality in young children, immunocompromised patients and the elderly, HuNoV disease causes substantial economic burden due to health care costs and loss of productivity [5,6,9,10,11]. A major barrier to studying HuNoVs for almost 50 years was the inability to develop robust and reproducible cultivation systems. Several reports of cultivation systems were not reproduced [12,13,14,15,16,17]. In addition, replication of a limited number of HuNoV strains has been reported in B cells [18,19]. For decades, a reproducible cultivation system for HuNoVs remained the gap required to be overcome to fully understand the biology and mechanisms that regulate the replication of these important pathogens. A cultivation system also is important to define HuNoV-host interactions that underlie the high virus infectivity and explosive illness they cause, to determine how to prevent transmission and to treat infection and illness. This chapter describes key advances that led to the development of a replication system in human intestinal epithelial cultures, applications of this system, and ongoing challenges to further optimize viral replication and understand HuNoV biology. Other chapters in this Special Issue of Viruses on *Caliciviridae* cover many aspects of the epidemiology, host susceptibility, molecular biology, immunity, and vaccine development as summarized by Desselberger [20]. 

## 2. Key Discoveries That Led to Replication of HuNoVs in Human Intestinal Enteroid Cultures

### 2.1. Early Attempts to Grow HuNoVs 

After HuNoVs were recognized as a cause of acute gastroenteritis and first characterized in 1972 [21], many attempts to cultivate these viruses in classical primary and immortalized cells were unsuccessful [22,23,24,25]. Several important lessons were learned from these early cultivation studies. For example, inoculation of cultured cells with stool filtrates requires careful vigilance and evaluation using important controls to be sure any morphologic cytopathic effects (CPE) detected are due to virus replication and not due to cytotoxicity caused by many non-viral toxic components in the stool. Controls can include treating filtrates with polymyxin B to negate the effects of lipopolysaccharides (LPS), using irradiated or heat-inactivated stools, and demonstrating that CPE or virus replication can be neutralized by virus capsid-specific polyclonal or monoclonal antibodies and not by pre-immune control sera or by an isotype control monoclonal antibody. It is also important to demonstrate that there is a net increase in virus titer in the culture system since virus that initially adheres to cells can subsequently be released back into the overlying media and surveillance of only the culture supernatant can lead to the erroneous conclusion that an increase of viral RNA in the media is due to virus replication rather than elution of initially bound virus from the surface of cells.

Classical cell lines used to cultivate many viruses were evaluated in early studies. In spite of some stools containing high levels of genomic copies of the virus and the evaluation of many conditions, including adding proteolytic enzymes required to grow other mucosal viruses, HuNoVs failed to replicate in vitro. This raised the following questions: (1) where does the virus replicate in infected persons and can that knowledge help predict what cell types support human HuNoV replication? and (2) are there clues from volunteer studies or studies of enteric calicivirus infections in animals that could help develop a system to cultivate the virus in vitro? 

**Figure 1:**
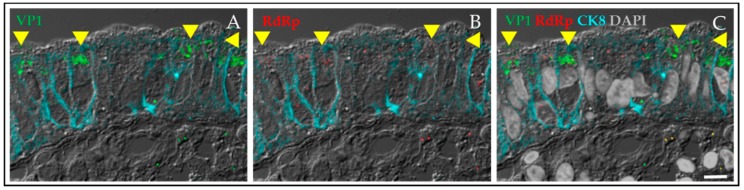
Human norovirus (HuNoV) replicates in enterocytes. Viral antigen was detected in a duodenal biopsy from an individual with common variable immune deficiency who was chronically infected with human norovirus. The biopsy was positive for HuNoV RNA. The viral capsid antigen VP1 in green (**A**) and the viral nonstructural protein, RNA-dependent RNA polymerase (RdRp) in red (**B**) were detected in enterocytes outlined by cyan staining for the epithelial marker cytokeratin 8 (CK8). The yellow arrows highlight cells expressing both the viral capsid antigen and the polymerase. There is little intestinal inflammation in this biopsy from a patient not on immunosuppressive therapy and few cells in the lamina propria show VP1 staining (**C**). Scale bar, 10 µm.

HuNoVs are members of the *Norovirus* genus of the *Caliciviridae* family. This virus family includes four other genera (*Lagovirus, Nebovirus, Sapovirus* and *Vesivirus*) and six other proposed genera [20]. The most notable genera in the family are *Norovirus* and *Sapovirus*, which cause severe acute gastroenteritis in humans and animals, with HuNoVs alone causing over 200,000 deaths annually in children <5 years of age [26]. The Cowden strain of porcine sapovirus (PSaV) was the first virus strain in the *Sapovirus* genus to be cultivated, and the addition of porcine intestinal content (IC) fluid filtrate was required for in vitro cultivation in primary porcine kidney cells [27,28]. Subsequent studies revealed that bile and bile acids, specifically glycochenodeoxycholic acid (GCDCA), function as active factors essential for PSaV growth in a continuous cell line (LLC-PK; [29]). In vivo studies in gnotobiotic piglets showed clear replication of HuNoV at early time points in enterocytes on the tips of small intestinal villi [30] and a similar pattern of replication was seen in gnotobiotic calves inoculated with a bovine norovirus [31] or a HuNoV strain [32]. At later time points, some HuNoV antigen was seen in the lamina propria. Volunteers inoculated with the prototypic HuNoV Norwalk virus showed morphologic changes in small intestinal biopsies [33,34,35,36]. Once specific antibodies to Norwalk virus-like particles (VLPs) were available, we identified viral antigen in enterocytes of duodenal biopsies from ill volunteers (this was not published at the time due to the limited number of biopsies examined). A subsequent study of biopsies from immunocompromised transplant patients detected viral antigen (both structural and nonstructural proteins) in enterocytes, and no HuNoV antigen staining was detected in the villus crypts. Some viral antigen also was present in the lamina propria where inflammation was prevalent [37]. Some nonstructural protein staining was seen in cells within the lamina propria and this staining was only in macrophages. The epithelial marker cytokeratin 8 (CK8) also was detected within these macrophages along with viral antigen [37], suggesting that virus was not replicating in those cells but rather that antigen in the lamina propria likely represented infected enterocytes phagocytosed by immune cells. This interpretation is consistent with the appearance of noroviral antigen in the lamina propria of gnotobiotic pigs and calves at later time points in infection. Staining of a biopsy from a patient with congenital variable immunoglobulin deficiency who is chronically infected with HuNoV detected viral capsid protein in enterocytes in the absence of significant immune cells in the lamina propria (Figure 1). Taken together, these data clearly indicate that HuNoVs replicate in intestinal enterocytes in humans. 

### 2.2. Key Discoveries That Helped Develop a Human Epithelial Cultivation System

Volunteer studies performed between 1972 and the 1990s recognized that a subset of individuals appeared to be genetically resistant to infection with Norwalk virus, and it was suggested that a host receptor might be missing in some people [38]. This was confirmed by the demonstration that Norwalk virus-like particles do not bind to intestinal biopsies of individuals called non-secretors who do not express the correct fucosylated glycans or histo-blood group antigens (HBGAs) on their intestinal villi because they lack a functional fucosyltransferase 2 (FUT2) enzyme [39]. This hypothesis was further confirmed by the discovery that only secretor-positive volunteers (persons with a functional FUT2 enzyme) were infected by the Norwalk virus in independent controlled human infection studies [40,41,42]. Taken together, these data led to the hypothesis that some HuNoVs would replicate in nontransformed human epithelial cultures obtained from secretor-positive donors where the correct HBGAs are expressed on enterocytes. This point is important because some cultured cells, such as the HK2 human kidney cells that are genotypically FUT2 positive do not express HBGAs [43]. 

For many years, cultivation of primary human intestinal epithelial cells was not possible because enterocytes, when directly cultured from human tissue, undergo apoptosis too rapidly to allow viral replication to be assessed. Progress in the fields of developmental and stem cell biology led to the ability to culture stem cell-derived epithelial only cultures (initially called organoids) from mouse and subsequently human intestinal surgical specimens or biopsies [44,45]. These intestinal stemcell-derived cultures also are called enteroids as recommended by the Intestinal Stem Cell Consortium [46] to distinguish them from intestinal organoid cultures that contain a mesenchymal niche and are produced from directed differentiation of embryonic or pluripotent stem cells [47]. The self-organizing, nontransformed mini-gut cultures are a remarkable new technology with many applications [48], including studying host-microbe interactions as illustrated by studies with HuNoVs, rotaviruses and other enteric microbes as reviewed elsewhere [49,50,51]. 

## 3. Components of the Human Intestinal Enteroid (HIE) Cultivation System 

Cultivation of HuNoVs requires stool samples containing infectious virus and enteroid cultures. We have established a bank of enteroid cultures primarily from adult donors. Detailed methods to produce new cultures and freeze down established cultures have been published [52,53,54] and are available upon request. Once established, these cultures can theoretically be propagated indefinitely, and many of our ongoing research projects on HuNoVs have used a jejunal line (J2) that was established in 2012. Maintenance of enteroid cultures requires a medium that contains growth factors to keep the stem cells growing. Trypsinized three-dimensional cultures are then seeded into collagen-coated 96-well plates to produce monolayers for infections. The culture medium is then changed to a medium without growth factors to stimulate the stem cells to differentiate into the distinct mature cell types such as enterocytes, goblet cells, Paneth cells, and enteroendocrine cells present in the epithelium. 

HuNoV infections are performed after 5 days of differentiation. For most experiments, we inoculate three wells of monolayers on a 96-well plate (technical replicates) for each time point and condition we are testing. Filtered stool suspensions are inoculated onto the monolayers. After a 1 h adsorption period, the inoculum is removed and the monolayers are washed to remove any unadsorbed virus. Three wells are then harvested to extract RNA to determine the baseline amount of virus present at 1 h and other triplicate wells are harvested at different time points to evaluate virus replication by RT-qPCR. Virus replication can also be assessed by other methods including flow cytometry to evaluate the percentage of infected cells, by immunofluorescence, by the detection of proteolytic processing of expressed proteins using Western blots, or by visualization of produced virus particles by electron microscopy [52]. These assays require higher amounts of stool inoculum compared to measuring RNA replication by RT-qPCR as the endpoint. The cultivation system has been disseminated and reproduced in a number of laboratories throughout the world and results from other laboratories are beginning to be published [55,56,57,58,59]. Below we highlight key features of the cultivation system and remaining challenges. 

## 4. Results of HuNoV Cultivation in HIEs 

### 4.1. Characteristics of Cultivation of HuNoV Strains in HIEs

#### 4.1.1. Multiple Strains Are Cultivatable and There Are Strain-Specific Requirements for Replication 

**Figure 2:**
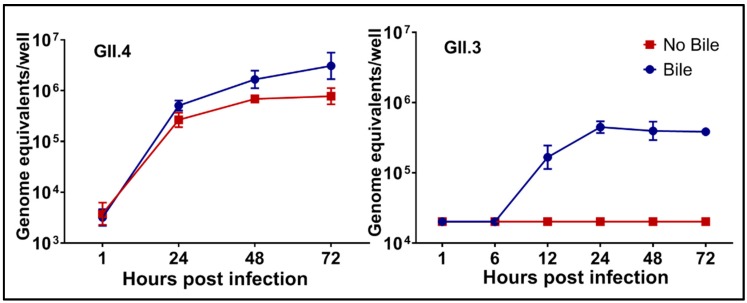
There are strain-specific requirements for human norovirus (HuNoV) replication. Replication of GII.4/Sydney/2012-like virus occurs in the absence of exogenously added human bile but it is enhanced by bile while bile is essential for replication of a GII.3 HuNoV strain. Virus growth curves show RNA replication can be detected by 12 h post-infection and replication plateaus by 48 h post-infection. Error bars denote standard deviation. (From [52]).

A variety of HuNoV strains have been successfully cultured in HIEs. Replication, as detected by increases in viral RNA, is seen by 12 h post-infection (hpi) but we routinely use 24 hpi to detect fold-increases in replication (Figure 2). Replication of a single GII.4 Sydney strain of HuNoV was reproducible in the HIE culture over more than a year [56] (and Estes unpublished data). A variety of virus strains including GI.1, GII.1, GII.2, GII.3, 7 variants of GII.4, and GII.17 have shown primary replication as assessed by RT-qPCR and replication of GII.6, GII.8, GII.12 and GII.14 has been detected by immunofluorescence. Most of our studies have compared replication of the GII.4/Sydney/2012-like virus and a GII.3 strain because early studies determined these viruses exhibit strain-specific requirements for replication that we continue to seek to understand. For example, the GII.3 virus failed to grow in the absence of exogenously added bile while the GII.4 strain grows without bile but replication is enhanced in the presence of bile (Figure 2).

#### 4.1.2. HuNoV Replication in Human Intestinal Enteroids Is Biologically Relevant 

Replication of HuNoVs in HIEs is biologically relevant based on the observation that the replication of GII.4 strains is restricted to cultures derived from secretor-positive persons while GII.3 strains replicate in secretor-positive cultures and a subset of secretor-negative cultures, mimicking the findings of epidemiological studies with these strains [60,61]. In early studies, the dose of inoculum required to produce infection in 50% of inoculated tissue culture wells (TCID_50_) was ~1.2 × 10^2^ genomic equivalents (ge) for GII.4/Sydney/2012 and ~2.0 × 10^4^ for GII.3. Similar results were obtained when a larger number of inocula were later tested; the TCID_50_ was 2.1 × 10^3^ ge/well for GII.4/Den Haag/2006b-like strain, 4.4 × 10^2^ for GII.4/Sydney/2012-like virus and 4.0 × 10^3^ for a GII.3 strain that were successfully cultivated [56]. These values are consistent with the human infectious dose 50 (HID_50_) of 1.3 × 10^3^ ge for secretor-positive blood group O or A persons and ~2.8 × 10^3^ for all secretor-positive persons as determined in a controlled human infection model with GI.1 Norwalk virus [62]. 

HuNoV replication requires differentiated HIE cultures that contain the multicellular types of a differentiated epithelium [44,53,54,63]. In these differentiated cultures, replication has only been detected by immunofluorescent staining in enterocytes, a result that contrasts with human rotavirus replication in these cultures, where the virus replicates in enterocytes and enteroendocrine cells [53,64]. Lack of HuNoV replication in proliferating, undifferentiated HIE cultures that primarily contain stem cells is consistent with the lack of detection of these viruses in the stem cells located in the crypts of infected persons or animals [30,31,37]. 

Limited passaging of GII.4 virus is possible but sustained passaging (after passage 4) remains to be achieved. However, the culture system produces infectious virus, as a laboratory researcher became infected with a passaged virus that was confirmed to be the same strain as the virus in culture. This experience is a reminder that HuNoVs are highly infectious and BSL2 laboratory procedures must be used at all times. 

#### 4.1.3. Characteristics of Inoculum Factors That Affect Successful Replication

Establishment of a new cultivation system requires significant optimization and determination of what factors affect successful replication. This remains an ongoing research project as the number and range of virus strains evaluated increases. Current information indicates bacterial-free fecal filtrates containing high titer virus replicate in HIEs, and successful replication depends on viral load and genotype of the inoculum [52,56]. From these published papers, in general, GII.4 viruses appear to have higher replication levels than other genotypes. Virus from stools collected from children and adults have replicated, but the frequency of successful cultivation is higher with stool inocula from children [56]; this may be due, in part, to higher virus titers in the samples from these children. When stratified by genotype, an effect of the initial input of virus was observed for multiple virus strains. Viral RNA replication from 1–6 days post-infection for viruses successfully cultivated by our laboratory and the CDC has ranged from approximately 10 to 1000-fold increases. Not all stool inocula with high virus titers grow in HIEs and while this is not surprising when cultivating viruses from clinical samples, further information is needed to understand whether other factors influence successful cultivation. It is possible that fecal specimens that do not replicate may not have been collected at an optimal early time after infection, may not have been stored optimally, or they may contain large numbers of noninfectious particles. Although precise optimal conditions for storing stool specimens remains to be established, all our stool specimens are stored long-term at −80 °C. Other unknown factors in the stool (concentrations of bile or other intestinal components) may also be relevant. In addition, HuNoV replication has only been tested in a limited number of HIE cultures from secretor positive donors (primarily J2 and J3 lines). Although GII.4/Sydney-like and GII.3 viruses can replicate in cultures from all three segments of the small intestine, testing cultures from more individuals may identify an enteroid line with increased susceptibility to more virus strains. It also is possible that the expression of other ligands besides secretor glycans are required for some currently non-cultivatable strains.

### 4.2. Applications of the Cultivation System

Although HuNoVs cause significant human disease, there are currently no licensed vaccines or antivirals for these pathogens. The previous inability to cultivate virus hampered the development of strategies to control and prevent HuNoV infection and to determine the effectiveness of methods to inactivate virus to prevent transmission in various settings, including in food or on contaminated surfaces. The persistence of HuNoVs in the environment, their high transmissibility, and the problem of chronic infection of immunocompromised individuals document a need for improved strategies for the treatment and prophylaxis of norovirus infections. 

The HIE culture system can be used to measure neutralizing antibodies. Initial studies found that virus-specific serum neutralization titers were higher compared to HBGA-blocking antibody titers, a previously described surrogate neutralization assay found to correlate with protection against clinical gastroenteritis in controlled human infection studies [52,65]. How neutralizing antibodies correlate with HBGA-blocking antibodies remains to be determined. Neutralization assays have also been used to characterize the first human monoclonal antibodies that neutralize pandemic GII.4 viruses [55] and a mouse monoclonal that targets the HBGA-binding pocket [57]. Future applications of neutralization assays should help define whether genotypes correlate with serotypes and whether neutralizing antibody responses are heterotypic. 

Previous evaluation of control measures for HuNoVs, including disinfection measures, relied primarily on the use of cultivatable surrogate viruses [66]. Using the new HIE cultivation system, it is now possible to directly evaluate methods of virus inactivation. Heat treatment at 60 °C for as little as 15 min and irradiation have been demonstrated to inactivate GII.4/Sydney-like and GII.3 viruses [52], and treatment with chlorine completely inactivated 3 GII.4 viruses at concentrations as low as 50 ppm of chlorine [56]. By contrast, regardless of concentration or exposure time, alcohols only slightly reduced and did not completely inactivate, HuNoV infectivity [56]. These results with alcohols and HuNoV inactivation resemble data from studies with Tulane virus [56,66]. Taken together, these studies confirm the utility of the HIE cultivation system as an important tool to study HuNoV inactivation strategies.

## 5. Discussion

Cultivation of multiple strains of HuNoVs in stem cell-derived HIEs is a long-awaited breakthrough for these important pathogens. These human cultures are transformative tools to study enteric viruses, and their use has identified unique host-viral interactions, cellular tropisms as well as innate and physiologic human epithelial responses to infection. For example, studies of cell tropism show that while HuNoVs infect enterocytes [37,52], rotaviruses infect enterocytes and enteroendocrine cells [53]. Likewise, a respiratory but not an enteric strain of adenovirus preferentially replicates in goblet cells [67], and echovirus 11 preferentially infects enteroendocrine cells [68]. These different tropisms may have physiologic consequences as illustrated for rotavirus infection of enteroendocrine cells; this stimulates serotonin secretion that is postulated to be important for the pathophysiologic vomiting response in children [53,64,69,70]. Other responses to infection remain to be fully described. Distribution of the HuNoV cultivation system is allowing multiple laboratories to begin to perform studies on virus replication and to allow research needed for further optimization of this system and to understand strain-specific requirements for replication. 

Several challenges remain to be solved to further enhance this intestinal epithelial cultivation system. First, the medium used to maintain the cultures is expensive and more complicated than standard unicellular cancer-derived tissue culture systems used for many classical virology studies. The expense and complexity of the media are based on the need to add several growth factors into the medium to maintain stem cell growth in proliferating cultures. Producing growth factors by growing three cell lines is time-consuming. It is now possible to consider alternative methods to obtain the growth factors (Wnt-3A, R-Spondin, and Noggin), including producing them from a single cell line [71,72] or using a commercial medium that is now produced by Stem Cell Technologies. While the commercial medium is convenient, it may be too expensive for extensive use by most virology laboratories. However, with increasing numbers of laboratories adopting these novel culture systems and the establishment of cores to maintain cultures for multiple investigators, use of HIEs is becoming easier. 

Second, sustained passaging of HuNoV strains remains to be fully established. We predict that this may be related to the cellular innate response induced by an infection that likely is responsible for the plateau in growth observed between 24 and 72 hpi [52]. An alternative possibility for seeing a plateau is that all susceptible cells are infected by 24–48 hpi. This is unlikely because the same kinetics of replication are observed in infections performed at high and low multiplicities of infection and susceptible cells remain available at the later time points. Genetic modification of the HIE stem cells may overcome this limitation, but making such modified cultures takes time because manipulations by transfection are not efficient, and selection and cultivation of cloned cultures is a slow process. 

Third, a question arises about whether it is possible to increase the sensitivity of the cultures to infection. Based on the ID_50_ values determined for current cultivatable strains, we routinely inoculate a well of enteroid cultures with a minimum of 1 × 10^5^ genome equivalents. As noted above, while this may seem like a high level of virus, it is not so high when considering the amount of virus needed to cause human infection and the levels of virus shed in the feces of infected persons. It may be that the stool filtrates contain noninfectious particles or that there are other factors in the stool that limit replication. 

Fourth, what is the basis for the strain-specific replication requirements? To date, GII.4/Sydney strains seem to replicate more efficiently than other strains, and they can replicate in the absence of bile while other strains such as GI.1, GII.3, and GII.17 require bile. We have identified two components in bile that support replication [73]. Are there sequence-specific motifs that correlate with cultivation efficiency? 

Fifth, will testing of additional cultures identify new HIE lines that are more susceptible to infection? This would not be surprising based on the range of host responses to infection. Sixth, it may be possible to increase replication by manipulating the cultures so that more enterocytes are produced in the cultures or by testing co-cultures with mesenchymal or vascular cells or the intestinal microbiome to more fully represent the intestinal milieu. Finally, identifying the cellular receptor(s) for HuNoVs may allow simplified systems to be established.

## 6. Conclusions 

It has been a long road from the discovery of HuNoVs as the first viruses to cause gastroenteritis in humans to being able to cultivate these pathogens in human intestinal epithelial cultures. This achievement was based on knowledge of genetically-encoded host susceptibility factors and the site and cell type(s) where the virus replicates in the small intestine. Animal models of infection, particularly with the porcine enteric sapovirus and bovine noroviruses, as well as studies in volunteers and chronically-infected patients, provided important information to build an understanding of how one might establish a replication system in non-transformed human cells. Cultivation in HIEs has brought on a new era for HuNoV research and has stimulated efforts to establish other models. Replication of HuNoV in zebrafish [74] and in organoids made from pluripotent stem cells [75] are recently described new models that may offer unique insights into HuNoV biology. The future seems bright for progress in understanding molecular mechanisms that regulate HuNoV replication and developing effective therapies and methods to control virus transmission. 

## References

[1] Pires S.M., Fischer-Walker C.L., Lanata C.F., Devleesschauwer B., Hall A.J., Kirk M.D., Duarte A.S., Black R.E., Angulo F.J. (2015). Aetiology-specific estimates of the global and regional incidence and mortality of diarrhoeal diseases commonly transmitted through food. PLoS ONE.

[2] Ramani S., Atmar R.L., Estes M.K. (2014). Epidemiology of human noroviruses and updates on vaccine development. Curr. Opin. Gastroenterol..

[3] Hall A.J., Lopman B.A., Payne D.C., Patel M.M., Gastanaduy P.A., Vinje J., Parashar U.D. (2013). Norovirus disease in the United States. Emerg. Infect. Dis..

[4] Ahmed S.M., Hall A.J., Robinson A.E., Verhoef L., Premkumar P., Parashar U.D., Koopmans M., Lopman B.A. (2014). Global prevalence of norovirus in cases of gastroenteritis: A systematic review and meta-analysis. Lancet. Infect. Dis..

[5] Havelaar A.H., Kirk M.D., Torgerson P.R., Gibb H.J., Hald T., Lake R.J., Praet N., Bellinger D.C., de Silva N.R., Gargouri N. (2015). World health organization global estimates and regional comparisons of the burden of foodborne disease in 2010. PLoS Med..

[6] Kirk M.D., Pires S.M., Black R.E., Caipo M., Crump J.A., Devleesschauwer B., Dopfer D., Fazil A., Fischer-Walker C.L., Hald T. (2015). Correction: World health organization estimates of the global and regional disease burden of 22 foodborne bacterial, protozoal, and viral diseases, 2010: A data synthesis. PLoS Med..

[7] Koo H.L., Neill F.H., Estes M.K., Munoz F.M., Cameron A., DuPont H.L., Atmar R.L. (2013). Noroviruses: The most common pediatric viral enteric pathogen at a large university hospital after introduction of rotavirus vaccination. J. Pediatric Infect. Dis. Soc..

[8] Payne D.C., Vinje J., Szilagyi P.G., Edwards K.M., Staat M.A., Weinberg G.A., Hall C.B., Chappell J., Bernstein D.I., Curns A.T. (2013). Norovirus and medically attended gastroenteritis in U.S. children. N. Engl. J. Med..

[9] Said M.A., Perl T.M., Sears C.L. (2008). Healthcare epidemiology: Gastrointestinal flu: Norovirus in health care and long-term care facilities. Clin. Infect. Dis..

[10] Bartsch S.M., Lopman B.A., Hall A.J., Parashar U.D., Lee B.Y. (2012). The potential economic value of a human norovirus vaccine for the United States. Vaccine.

[11] Bartsch S.M., Lopman B.A., Ozawa S., Hall A.J., Lee B.Y. (2016). Global economic burden of norovirus gastroenteritis. PLoS ONE.

[12] Herbst-Kralovetz M.M., Radtke A.L., Lay M.K., Hjelm B.E., Bolick A.N., Sarker S.S., Atmar R.L., Kingsley D.H., Arntzen C.J., Estes M.K. (2013). Lack of norovirus replication and histo-blood group antigen expression in 3-dimensional intestinal epithelial cells. Emerg. Infect. Dis..

[13] Papafragkou E., Hewitt J., Park G.W., Greening G., Vinje J. (2014). Challenges of culturing human norovirus in three-dimensional organoid intestinal cell culture models. PLoS ONE.

[14] Straub T.M., Bartholomew R.A., Valdez C.O., Valentine N.B., Dohnalkova A., Ozanich R.M., Bruckner-Lea C.J., Call D.R. (2011). Human norovirus infection of Caco-2 cells grown as a three-dimensional tissue structure. J. Water Health.

[15] Straub T.M., Honer zu Bentrup K., Orosz-Coghlan P., Dohnalkova A., Mayer B.K., Bartholomew R.A., Valdez C.O., Bruckner-Lea C.J., Gerba C.P., Abbaszadegan M. (2007). In vitro cell culture infectivity assay for human noroviruses. Emerg. Infect. Dis..

[16] Straub T.M., Hutchison J.R., Bartholomew R.A., Valdez C.O., Valentine N.B., Dohnalkova A., Ozanich R.M., Bruckner-Lea C.J. (2013). Defining cell culture conditions to improve human norovirus infectivity assays. Water Sci. Technol..

[17] Takanashi S., Saif L.J., Hughes J.H., Meulia T., Jung K., Scheuer K.A., Wang Q. (2014). Failure of propagation of human norovirus in intestinal epithelial cells with microvilli grown in three-dimensional cultures. Arch. Virol..

[18] Jones M.K., Watanabe M., Zhu S., Graves C.L., Keyes L.R., Grau K.R., Gonzalez-Hernandez M.B., Iovine N.M., Wobus C.E., Vinje J. (2014). Enteric bacteria promote human and mouse norovirus infection of B cells. Science.

[19] Bhar S., Jones M.K. (2019). In vitro replication of human norovirus. Viruses.

[20] Desselberger U. (2019). Caliciviridae other than noroviruses. Viruses.

[21] Kapikian A.Z., Wyatt R.G., Dolin R., Thornhill T.S., Kalica A.R., Chanock R.M. (1972). Visualization by immune electron microscopy of a 27-nm particle associated with acute infectious nonbacterial gastroenteritis. J. Virol..

[22] Duizer E., van Duynhoven Y., Vennema H., Koopmans M. (2004). Failure to detect norovirus in a large group of asymptomatic individuals by Marshall et al. (Public Health vol 118 (3) 230-233). Public Health.

[23] Kapikian A.Z. (2000). The discovery of the 27-nm Norwalk virus: An historic perspective. J. Infect. Dis..

[24] Lay M.K., Atmar R.L., Guix S., Bharadwaj U., He H., Neill F.H., Sastry K.J., Yao Q., Estes M.K. (2010). Norwalk virus does not replicate in human macrophages or dendritic cells derived from the peripheral blood of susceptible humans. Virology.

[25] Moore M.D., Goulter R.M., Jaykus L.A. (2015). Human norovirus as a foodborne pathogen: Challenges and developments. Annu. Rev. Food Sci. Technol..

[26] Atmar R.L., Ramani S., Estes M.K. (2018). Human noroviruses: Recent advances in a 50-year history. Curr. Opin. Infect. Dis..

[27] Flynn W.T., Saif L.J. (1988). Serial propagation of porcine enteric calicivirus-like virus in primary porcine kidney cell cultures. J. Clin. Microbiol..

[28] Parwani A.V., Flynn W.T., Gadfield K.L., Saif L.J. (1991). Serial propagation of porcine enteric calicivirus in a continuous cell line. Effect of medium supplementation with intestinal contents or enzymes. Arch. Virol..

[29] Chang K.O., Sosnovtsev S.V., Belliot G., Kim Y., Saif L.J., Green K.Y. (2004). Bile acids are essential for porcine enteric calicivirus replication in association with down-regulation of signal transducer and activator of transcription 1. Proc. Natl. Acad. Sci. USA.

[30] Cheetham S., Souza M., Meulia T., Grimes S., Han M.G., Saif L.J. (2006). Pathogenesis of a genogroup II human norovirus in gnotobiotic pigs. J. Virol..

[31] Otto P.H., Clarke I.N., Lambden P.R., Salim O., Reetz J., Liebler-Tenorio E.M. (2011). Infection of calves with bovine norovirus GIII.1 strain Jena virus: An experimental model to study the pathogenesis of norovirus infection. J. Virol..

[32] Souza M., Azevedo M.S., Jung K., Cheetham S., Saif L.J. (2008). Pathogenesis and immune responses in gnotobiotic calves after infection with the genogroup II.4-HS66 strain of human norovirus. J. Virol..

[33] Agus S.G., Dolin R., Wyatt R.G., Tousimis A.J., Northrup R.S. (1973). Acute infectious nonbacterial gastroenteritis: Intestinal histopathology. Histologic and enzymatic alterations during illness produced by the Norwalk agent in man. Ann. Intern. Med..

[34] Dolin R., Levy A.G., Wyatt R.G., Thornhill T.S., Gardner J.D. (1975). Viral gastroenteritis induced by the Hawaii agent. Jejunal histopathology and serologic response. Am. J. Med..

[35] Schreiber D.S., Blacklow N.R., Trier J.S. (1973). The mucosal lesion of the proximal small intestine in acute infectious nonbacterial gastroenteritis. N. Engl. J. Med..

[36] Schreiber D.S., Blacklow N.R., Trier J.S. (1974). The small intestinal lesion induced by Hawaii agent acute infectious nonbacterial gastroenteritis. J. Infect. Dis..

[37] Karandikar U.C., Crawford S.E., Ajami N.J., Murakami K., Kou B., Ettayebi K., Papanicolaou G.A., Jongwutiwes U., Perales M.A., Shia J. (2016). Detection of human norovirus in intestinal biopsies from immunocompromised transplant patients. J. Gen. Virol..

[38] Parrino T.A., Schreiber D.S., Trier J.S., Kapikian A.Z., Blacklow N.R. (1977). Clinical immunity in acute gastroenteritis caused by Norwalk agent. N. Engl. J. Med..

[39] Marionneau S., Ruvoen N., Le Moullac-Vaidye B., Clement M., Cailleau-Thomas A., Ruiz-Palacois G., Huang P., Jiang X., Le Pendu J. (2002). Norwalk virus binds to histo-blood group antigens present on gastroduodenal epithelial cells of secretor individuals. Gastroenterology.

[40] Hutson A.M., Airaud F., LePendu J., Estes M.K., Atmar R.L. (2005). Norwalk virus infection associates with secretor status genotyped from sera. J. Med. Virol..

[41] Hutson A.M., Atmar R.L., Graham D.Y., Estes M.K. (2002). Norwalk virus infection and disease is associated with ABO histo-blood group type. J. Infect. Dis..

[42] Lindesmith L., Moe C., Marionneau S., Ruvoen N., Jiang X., Lindblad L., Stewart P., LePendu J., Baric R. (2003). Human susceptibility and resistance to Norwalk virus infection. Nat. Med..

[43] Ravn V., Dabelsteen E. (2000). Tissue distribution of histo-blood group antigens. Apmis.

[44] Sato T., Stange D.E., Ferrante M., Vries R.G., Van Es J.H., Van den Brink S., Van Houdt W.J., Pronk A., Van Gorp J., Siersema P.D. (2011). Long-term expansion of epithelial organoids from human colon, adenoma, adenocarcinoma, and Barrett’s epithelium. Gastroenterology.

[45] Sato T., Vries R.G., Snippert H.J., van de Wetering M., Barker N., Stange D.E., van Es J.H., Abo A., Kujala P., Peters P.J. (2009). Single LGR5 stem cells build crypt-villus structures in vitro without a mesenchymal niche. Nature.

[46] Stelzner M., Helmrath M., Dunn J.C., Henning S.J., Houchen C.W., Kuo C., Lynch J., Li L., Magness S.T., Martin M.G. (2012). A nomenclature for intestinal in vitro cultures. Am. J. Physiol. Gastrointest. Liver Physiol..

[47] Spence J.R., Mayhew C.N., Rankin S.A., Kuhar M.F., Vallance J.E., Tolle K., Hoskins E.E., Kalinichenko V.V., Wells S.I., Zorn A.M. (2011). Directed differentiation of human pluripotent stem cells into intestinal tissue in vitro. Nature.

[48] Sato T., Clevers H. (2013). Growing self-organizing mini-guts from a single intestinal stem cell: Mechanism and applications. Science.

[49] Blutt S.E., Crawford S.E., Ramani S., Zou W.Y., Estes M.K. (2018). Engineered human gastrointestinal cultures to study the microbiome and infectious diseases. Cell Mol. Gastroenterol. Hepatol..

[50] Dutta D., Clevers H. (2017). Organoid culture systems to study host-pathogen interactions. Curr. Opin. Immunol..

[51] Ramani S., Crawford S.E., Blutt S.E., Estes M.K. (2018). Human organoid cultures: Transformative new tools for human virus studies. Curr. Opin. Virol..

[52] Ettayebi K., Crawford S.E., Murakami K., Broughman J.R., Karandikar U., Tenge V.R., Neill F.H., Blutt S.E., Zeng X.L., Qu L. (2016). Replication of human noroviruses in stem cell-derived human enteroids. Science.

[53] Saxena K., Blutt S.E., Ettayebi K., Zeng X.L., Broughman J.R., Crawford S.E., Karandikar U.C., Sastri N.P., Conner M.E., Opekun A.R. (2016). Human intestinal enteroids: A new model to study human rotavirus infection, host restriction, and pathophysiology. J. Virol..

[54] Zou W.Y., Blutt S.E., Crawford S.E., Ettayebi K., Zeng X.L., Saxena K., Ramani S., Karandikar U.C., Zachos N.C., Estes M.K. (2017). Human intestinal enteroids: New models to study gastrointestinal virus infections. Methods Mol. Biol..

[55] Alvarado G., Ettayebi K., Atmar R.L., Bombardi R.G., Kose N., Estes M.K., Crowe J.E. (2018). Human monoclonal antibodies that neutralize pandemic GII.4 noroviruses. Gastroenterology.

[56] Costantini V., Morantz E.K., Browne H., Ettayebi K., Zeng X.L., Atmar R.L., Estes M.K., Vinje J. (2018). Human norovirus replication in human intestinal enteroids as model to evaluate virus inactivation. Emerg. Infect. Dis..

[57] Koromyslova A.D., Morozov V.A., Hefele L., Hansman G.S. (2019). Human norovirus neutralized by a monoclonal antibody targeting the histo-blood group antigen pocket. J. Virol..

[58] Lindesmith L.C., McDaniel J.R., Changela A., Verardi R., Kerr S.A., Costantini V., Brewer-Jensen P.D., Mallory M.L., Voss W.N., Boutz D.R. (2019). Sera antibody repertoire analyses reveal mechanisms of broad and pandemic strain neutralizing responses after human norovirus vaccination. Immunity.

[59] Chan M.C.W., Cheung S.K.C., Mohammad K.N., Chan J.C.M., Estes M.K., Chan P.K.S. (2019). Use of human intestinal enteroids to correlate nucleic acid test with human norovirus infectivity. Emerg. Infect. Dis..

[60] Ruvoen-Clouet N., Belliot G., Le Pendu J. (2013). Noroviruses and histo-blood groups: The impact of common host genetic polymorphisms on virus transmission and evolution. Rev. Med. Virol..

[61] Ayouni S., Estienney M., Sdiri-Loulizi K., Ambert-Balay K., de Rougemont A., Aho S., Hammami S., Aouni M., Guediche M.N., Pothier P. (2015). Relationship between GII.3 norovirus infections and blood group antigens in young children in Tunisia. Clin. Microbiol. Infect..

[62] Atmar R.L., Opekun A.R., Gilger M.A., Estes M.K., Crawford S.E., Neill F.H., Ramani S., Hill H., Ferreira J., Graham D.Y. (2014). Determination of the 50% human infectious dose for Norwalk virus. J. Infect. Dis..

[63] Fujii M., Matano M., Toshimitsu K., Takano A., Mikami Y., Nishikori S., Sugimoto S., Sato T. (2018). Human intestinal organoids maintain self-renewal capacity and cellular diversity in niche-inspired culture condition. Cell Stem Cell.

[64] Chang-Graham A.L., Danhof H.A., Engevik M.A., Tomaro-Duchesneau C., Karandikar U.C., Estes M.K., Versalovic J., Britton R.A., Hyser J.M. (2019). Human intestinal enteroids with inducible neurogenin-3 expression as a novel model of gut hormone secretion. Cell Mol. Gastroenterol. Hepatol..

[65] Reeck A., Kavanagh O., Estes M.K., Opekun A.R., Gilger M.A., Graham D.Y., Atmar R.L. (2010). Serological correlate of protection against norovirus-induced gastroenteritis. J. Infect. Dis..

[66] Cromeans T., Park G.W., Costantini V., Lee D., Wang Q., Farkas T., Lee A., Vinje J. (2014). Comprehensive comparison of cultivable norovirus surrogates in response to different inactivation and disinfection treatments. Appl. Environ. Microbiol..

[67] Holly M.K., Smith J.G. (2018). Paneth cells during viral infection and pathogenesis. Viruses.

[68] Drummond C.G., Bolock A.M., Ma C., Luke C.J., Good M., Coyne C.B. (2017). Enteroviruses infect human enteroids and induce antiviral signaling in a cell lineage-specific manner. Proc. Natl. Acad. Sci. USA.

[69] Hagbom M., Istrate C., Engblom D., Karlsson T., Rodriguez-Diaz J., Buesa J., Taylor J.A., Loitto V.M., Magnusson K.E., Ahlman H. (2011). Rotavirus stimulates release of serotonin (5-HT) from human enterochromaffin cells and activates brain structures involved in nausea and vomiting. PLoS Pathog..

[70] Hagbom M., Sharma S., Lundgren O., Svensson L. (2012). Towards a human rotavirus disease model. Curr. Opin. Virol..

[71] VanDussen K.L., Marinshaw J.M., Shaikh N., Miyoshi H., Moon C., Tarr P.I., Ciorba M.A., Stappenbeck T.S. (2015). Development of an enhanced human gastrointestinal epithelial culture system to facilitate patient-based assays. Gut.

[72] VanDussen K.L., Sonnek N.M., Stappenbeck T.S. (2019). L-WRN conditioned medium for gastrointestinal epithelial stem cell culture shows replicable batch-to-batch activity levels across multiple research teams. Stem Cell Res..

[73] Murakami K., Tenge V.R., Karandikar U., Lin S.C., Ramani S., Ettayebi K., Crawford S.E., Zeng X.L., Neill F.H., Ayyar B.V. (2019). Bile acids and ceramide overcome the entry restriction for GII.3 human norovirus replication in human intestinal enteroids.

[74] Van Dycke J., Ny A., Conceicao-Neto N., Maes J., Hosmillo M., Cuvry A., Goodfellow I., de Araujo Nogueira T.C., Verbeken E., Matthijnssens J. (2019). A robust human norovirus replication model in zebrafish larvae. BioRxiv.

[75] Sato S., Hisaie K., Kurokawa S., Suzuki A., Sakon N., Uchida Y., Yuki Y., Kiyono H. (2019). Human norovirus propagation in human induced pluripotent stem cell-derived intestinal epithelial cells. Cell Mol. Gastroenterol. Hepatol..

